# Survival and Success Rates of Different Shoulder Designs: A Systematic Review of the Literature

**DOI:** 10.1155/2018/6812875

**Published:** 2018-04-26

**Authors:** Marco Tallarico, Marco Caneva, Silvio Mario Meloni, Erta Xhanari, Yuki Omori, Luigi Canullo

**Affiliations:** ^1^Implantology and Prosthetic Aspects, Master of Science in Dentistry Program, Aldent University, Tirana, Albania; ^2^Private Practice, Rome, Italy; ^3^ARDEC Academy, Ariminum Odontologica Srl, Rimini, Italy; ^4^Dentistry Unit, University Hospital of Sassari, Sassari, Italy; ^5^Private Practice, Tirana, Albania; ^6^Department of Oral Implantology, Osaka Dental University, Hirakata, Osaka, Japan

## Abstract

**Objectives:**

To identify whether there is a relationship between different implant shoulder positions/orientations/designs and prosthetic and/or implant failures, biological or mechanical complications, radiographic marginal bone loss (MBL), peri-implant buccal recession (RC), aesthetic scores (Papilla Index, PES, and WES), and patient satisfaction after a minimum of 1 year function in the aesthetic zone, compared to the two-piece, conventional implant neck architecture.

**Materials and Methods:**

The systematic review was written according to the PRISMA guidelines. The search strategy encompassed the English literature from 1967 to September 2016 and was performed online (in the PubMed database of the U.S. National Library of Medicine, Embase, and the Cochrane Library) to identify relevant studies that met the inclusion criteria. The assessment of quality and risk of bias of the selected manuscripts was performed according to the guidelines provided by CONSORT and STROBE statements.

**Results:**

A total of 16 articles (7 randomized controlled trials, 4 observational comparative studies, and 5 systematic reviews) were selected to fulfill the inclusion criteria. A trend of higher implant failure and prosthetic complications were experienced in the one-piece group compared to the two-piece group, although no statistically significant differences were found. Higher marginal bone loss was found in the test group (one-piece, scalloped implants) compared to the control group (two-piece, flat implants). No comparative studies reporting data on sloped implants were found that fulfilled the inclusion and exclusion criteria of this systematic review. No differences were experienced between groups regarding aesthetic outcomes and patient satisfaction.

**Conclusions:**

There was sufficient evidence that different implant shoulder positions/orientations/designs (scalloped, sloped, and one piece) offer no benefit when compared to two-piece, conventional flat implants. Current evidence is limited due to the quality of available studies.

## 1. Introduction

Stability of the peri-implant soft and hard tissues is a prerequisite for a long-term aesthetic and function of implant-supported restoration [1]. In two-piece implants, early bone loss is observed after the connection of the abutment and delivery of final prosthesis, mostly due to the biologic width establishment [2–6]. This concept is being hypothesized as one of the most likely causes of early implant bone loss [2, 3]. The effect of surgical trauma, implant surface characteristics, macrodesign of the implant, and type of implant-abutment connection, as well as implant placement depth, soft tissue thickness, distance between adjacent implants, and abutment height, may all contribute to this process [4–6].

Traditionally, implants are two pieces, and they were placed in a two-step surgical procedure [7]. Two-piece designs can offer increased flexibility, with connections possible at the bone level, and wound closure can be easier.

In the 1980s [8], Schroeder and colleagues introduced an implant where the bone anchorage unit and contiguous transmucosal component were manufactured in a single unit. With one-piece implant designs, the transmucosal part is incorporated into the implants. This was an attempt to minimize crestal bone loss that reduces contamination of the implant-abutment junction. Furthermore, by using a one-piece implant, the second surgery procedure is avoided, as well as abutment connection/disconnection. The advantage of this procedure is to avoid the presence of a gap or micromovement at the implant-abutment junction for a beneficial effect on the peri-implant soft and hard tissues [9]. Nevertheless, compared with two-piece implants, they are much more difficult to place in the prosthetically driven position (height and angulation), which makes one-piece implants even more difficult to finalize. On the other hand, new implant and abutment designs have been proposed to minimize the crestal bone loss. Platform switching is done whenever an abutment is used that is smaller in diameter than the implant platform. This concept has been proposed as an effective prosthetic concept to reduce the amount of peri-implant bone loss around dental implants [10]. The concept of horizontal offset (platform switching) has made it possible to place implant shoulders at the crestal bone level with predictable minimal marginal bone resorption [10]. Scalloped and sloped implants represent other design changes that advocate for maintaining marginal bone levels [11–13]. The scalloped implant was designed with a modified platform that mirrors the natural cement-enamel junction of the anterior teeth and follows the anatomic contour of the anterior alveolar bone crest. The scalloped implants were developed with the intent of preserving interdental bony peaks, supporting the soft tissue, thereby maintaining or creating interimplant papillae [11, 12]. Recently, a dental implant with a sloped marginal contour and a height difference of the implant shoulder of approximately 1.5 mm has been developed with the aim of improving the congruence between the implant and the bone in extraction sites and sloped ridges [13].

The main objective of this systematic review was to compare the prosthetic and/or implant failures, biological or mechanical complications, radiographic marginal bone loss, peri-implant buccal recession, aesthetic scores, and patient satisfaction after at least 1 year of function, around single- or multiple-tooth implant-supported restorations in the aesthetic zone, on the two-piece, conventional implant neck architecture (flat implants with the same level on 360°) and one-piece, scalloped, or sloped implants.

## 2. Materials and Methods

This systematic review was written according to the Preferred Reporting Items for Systematic Reviews and Meta-Analyses (PRISMA) guidelines [14]. The focused question was to identify whether there is a relationship between different implant shoulder positions/orientations/designs (one piece, scalloped, and sloped) and prosthetic and/or implant failures, biological or mechanical complications, radiographic marginal bone loss, peri-implant buccal recession, aesthetic scores, and patient satisfaction after at least 1 year of function, compared to two-piece, flat implants with the same level on 360°. Initially, PICOS question (population (P), intervention (I), comparison (C), outcomes and study design (O), and study type (S)) defined the search strategy, where P = single and partial edentulous patients required an implant-supported restoration in the aesthetic zone; I = different implant shoulder positions/orientations/designs (scalloped, sloped, and one piece), after at least 1 year of function; C = two piece and same level on 360° (flat implants); O = prosthetic and/or implant failures, biological or mechanical complications, radiographic marginal bone loss (MBL), peri-implant buccal recession (BR), aesthetic scores (Papilla Index, PES, and WES), and patient satisfaction (patient questionnaire and VAS); and S = randomized controlled clinical trials (RCTs), case-control studies, and cohort studies.

### 2.1. Search Strategy

An initial search strategy that includes the English literature from 1967 to September 2016 was performed to identify relevant studies that met the inclusion criteria of this systematic review. The following databases were consulted: Embase (Excerpta Medica dataBASE), PubMed database of the U.S. National Library of Medicine, Grey Literature Database (New York Academy of Medicine Grey Literature Report), and the Cochrane Library. Screening was performed independently and simultaneously by two calibrated examiners (MT and SMM). The electronic databases were searched using the following terms: (((“dental implants”[Mesh] AND “dental implant abutment design”[Mesh]) OR “dental implant abutment interface”[All Fields]) OR (one[All Fields] AND piece[All Fields] AND implant[All Fields])) OR ((“scalloped”[All Fields]) AND implant[All Fields]) OR (sloped[All Fields] AND implant[All Fields]) AND English[lang].

### 2.2. Eligibility Criteria

The following inclusion criteria were defined for the selection of articles:

- Written in English
- Evaluate in their protocol the prosthetic and/or implant failures, biological or mechanical complications, radiographic marginal bone loss, peri-implant buccal recession, aesthetic scores, patient satisfaction, and/or the influence of the implant shoulder position/orientation/design on soft and hard tissue levels around single or multiple implants in the aesthetic zone with scalloped, sloped, and one-piece implants and a two-piece, conventional implant neck architecture (flat implants featured with the same level on 360°)
- Randomized controlled clinical trials of implants of ≥1 year in function
- Observational (prospective and retrospective) case-control studies of implants of ≥1 year in function
- Cross-sectional comparative studies of ≥1 year in function
- Systematic reviews, narrative reviews, consensus statements, commentaries, or editorials

Articles were excluded if they were

- observational (prospective or retrospective) cohort studies without the control group;
- in vitro studies;
- finite element analyses;
- animal studies;
- reports with <5 cases;
- reports involving mini-implants, zirconia implants, or blade implants;
- reports on implants of <1 year in function.

### 2.3. Data Extraction

The two calibrated reviewers screened all the data from the selected papers. Cohen's kappa values between examiners were calculated at both the stages of the research. Discrepancies were resolved by consensus, and a third examiner was consulted (LC). Articles without abstracts but with titles related to the objectives of this study were included, and their full texts were screened for possible eligibility. Reference lists of the selected articles, including published systematic reviews, were screened for possible additional papers.

The following outcome measures were analyzed when available: [1] prosthetic and/or implant failures leading to loss or removal of the prosthesis and/or implant [2], biological or mechanical complications [3], radiographic marginal bone loss (MBL) [4], peri-implant buccal recession (BR) [5], Papilla Index, pink aesthetic score (PES), and white aesthetic score (WES) [6], and patient satisfaction (patient questionnaire and VAS).

### 2.4. Assessment of Quality, Heterogeneity, and Risk of Bias of Individual Studies

The same reviewers assessed the quality of the included manuscripts, heterogeneity, and the risk of bias according to the guidelines provided by the CONSORT statement for the evaluation of randomized controlled trials (http://www.consort-statement.org) [15] and the STROBE statement for observational studies (http://www.strobe-statement.org), as well as the modified items from the Cochrane Collaboration's tool for assessing risk of bias [16]. The overall risk of bias was expressed as the percentage of negatively graded items [16]. Quality assessment was performed on the published full-text articles, independently by both reviewers. Disagreements between them were resolved upon discussion. An estimation of plausible risk of bias (low, moderate, or high) was completed for each selected study according to the Cochrane Handbook for Systematic Reviews of Interventions (version 5.1.0. http://www.cochrane.org/resources/handbook).

## 3. Results

### 3.1. Study Selection

**Figure 1:**
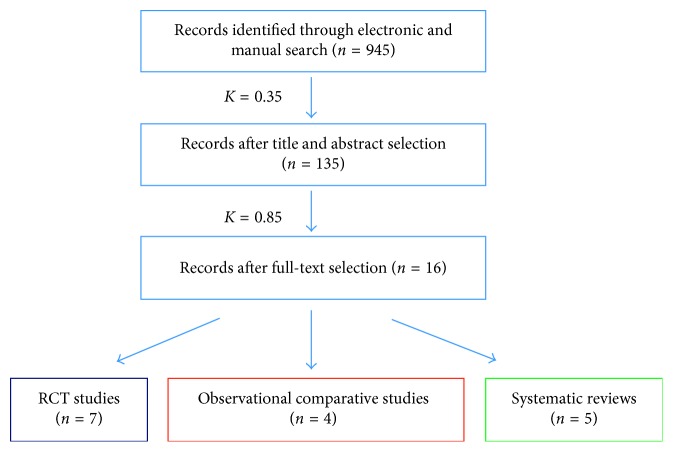
Flow chart.

A total of 945 potentially relevant titles and abstracts were found after the initial electronic and manual search. At this stage, 810 articles were excluded (% of agreement: 89.2%; Cohen's *k*: 0.35). Complete full-text manuscripts of the remaining 135 articles were evaluated, and 119 articles were excluded since they did not fulfill the inclusion criteria (% of agreement: 97.0; Cohen's *k*: 0.85), scoring an almost perfect agreement. Finally, a total of 16 articles that fulfilled the inclusion criteria of this systematic review were included in the qualitative analysis. Overall, data from 221 one-piece implants placed in 107 patients, 139 scalloped implants placed in 96 patients, and 366 flat implants (same level on 360°) placed in 207 patients were evaluated. No comparative studies reporting data on sloped implants that fulfilled the inclusion criteria were founded. Of the 16 selected studies, 7 were randomized controlled trials [9, 17–22], 4 were observational comparative studies (2 retrospective and 2 prospective) [23–26], and 5 were systematic reviews [27–31]. A diagram of the search strategy is shown in Figure 1.

Three pairs of manuscripts reported data from the same cohort of patients. Sanz Martin et al. [17] and Thoma et al. [18] published two manuscripts based on the same cohort of 60 patients, reporting volumetric soft tissue change and demographic and radiographic results, respectively. Van Nimwegen et al. [19] published a 5-year follow-up report on the 1-year report of Tymstra et al. [22]. Finally, den Hartog et al. published two manuscripts reporting data from single implants in the aesthetic zone with different neck designs. The first manuscript was published in 2011 and was aimed at reporting radiological and clinical outcome measures [20], while the second manuscript, published 2 years later, focused on the aesthetic outcomes from both professional's and patient's perception [21].

### 3.2. Risk of Bias

The 16 selected studies were published between 1993 and September 2016. None of the publications were associated with a low risk of bias, while five with a high risk of bias and six with a moderate risk of bias (Table 1). The included articles received minimum grading when written in agreement with the CONSORT/STROBE statement guidelines (0/11), evaluating submission to ethical committees (5/11), none or unclear randomization procedures (7/11), none or unclear allocation concealment (9/11), and blinding of participants/outcome assessors (0/11) (Table 1).

### 3.3. Prosthetic and/or Implant Failures and Biological or Mechanical Complications

Nine of the eleven clinical studies reported data on implant failure/success. Two studies regarding one-piece implants compared with two-piece implants scored a cumulative survival rate of 100% in both test and control groups [23, 26]. Thoma et al. [18] reported one implant failure in the one-piece group and none in the two-piece group. Duda et al. [24] reported 9 implant failures in the one-piece group (7 immediately loaded and 2 delayed loaded), while no implant failure was reported in the two-piece group. Most of these failures were experienced due to biological complications (peri-implantitis and lack of osseointegration). Conversely, Heijdenrijk et al. [9] reported 2 implant failures in the two-piece implants compared to no failure in the one-piece group. All the implant failures were reported within the first year after function.

Three studies reported data from scalloped implants. den Hartog et al. [20, 21] reported only 1 implant failure in the control group (same level on 360°) compared with scalloped implants. Tymstra et al. [22] reported a cumulative survival rate of 100% in both groups at 1 year of follow-up, while Van Nimwegen et al. [19], over the same cohort of patients, reported 2 implant failures in the scalloped group, 4 years after their placement, due to extensive peri-implant bone loss.

Only one study included clinical complications as an outcome measure [23]. In this study, Ormianer et al. reported 8 porcelain fractures in the two-piece group and 4 in the one-piece group. Nevertheless, no statistically significant difference was found. All data are reported in Table 2. Finally, two systematic reviews of Barrachina-Díez et al. [27, 28] reported a high long-term survival rate but also high frequency of technical and biological complications in one-piece implants, both in one-part and two-part designs.

### 3.4. One-Piece versus Two-Piece Implants (MBL and BR)

Sanz Martin et al. [17] and Thoma et al. [18] published two randomized controlled clinical trials on the same cohort of 60 patients (151 implants), aimed at assess the volumetric changes of the buccal soft tissues between baseline and 1 year after loading follow-up, [17] and to compare the clinical and radiographic outcomes using one-piece (*n*=65; Straumann) and two-piece (*n*=86; Nobel Biocare External Hex) dental implant systems [18]. These researches failed to find significant differences between the one- and two-piece implant types with regard to tissue thickness, crown height (CHC), and facial tissue volume (VC). Conversely, the two-piece group exhibited slightly less bone loss during the evaluated period. Differences between the two groups reached a statistical significance with less bone loss for the two-piece group.

Ormianer et al. [23] analyzed retrospectively one-piece (*n*=34; Zimmer One-Piece, Zimmer Biomet) and two-piece (*n*=38; Tapered Screw-Vent, Zimmer Biomet) implants placed in the mandible of the same patients (*n*=24) according to a split-mouth design. After 5 years of function, marginal bone loss did not significantly differ between one- and two-piece dental implant systems (the mean MBL is not reported).

Duda et al. [24] in a retrospective comparative study evaluated clinical outcomes of immediate insertion and loading of one-piece implants (49 implants in 13 patients; Q-Implant; Trinon Titanium GmbH, Karlsruhe, Germany), compared to delayed loading of immediately placed one-piece implants (24 implants in 11 patients; Q-Implant; Trinon Titanium GmbH), and delayed placed two-piece nonsubmerged implants (39 implants in 10 patients; Q-Implant; Trinon Titanium GmbH). Mean MBL was 1.45 mm and 1.71 mm at the 5-year follow-up for one-piece implants with immediate loading and delayed loading, respectively. In case of two-piece implants, the mean MBL was 0.9 mm at the 3-year follow-up. The authors concluded that two-piece implants showed less MBL compared with one-piece implants in both the maxilla and mandible [24]. On the other hand, there was no statistical difference in MBL between immediate and delayed loaded one-piece implants, but immediate loaded implants showed more MBL in the maxilla [24].

Finally, Heijdenrijk et al. [9] in a randomized controlled trial with 5-year follow-up reported that the microgap at the implant-abutment interface in two-piece implants does not appear to have an adverse effect on the amount of peri-implant bone loss compared with one-piece implants [9]. All of the data are reported in Table 2.

### 3.5. Scalloped Implants (MBL and BR)

Van Nimwegen et al. [19] randomly compared 20 patients with two adjacent implant-supported restorations delivered on scalloped implants (*n*=20; NobelPerfect Groovy, Nobel Biocare) and implants with a flat platform (*n*=20; NobelPerfect Groovy). This study is a 5-year follow-up on the 1-year preliminary report of Tymstra et al. [22]. More bone loss and more BoP with compromised interimplant papilla regeneration were found around scalloped implants. Nevertheless, the implant crown aesthetic index, as well as patient satisfaction, was not significantly different between the groups [19, 22].

den Hartog et al., in two similar randomized controlled trials with 18 months of follow-up [20, 21], evaluated the aesthetic outcome and the marginal bone level changes of anterior single-tooth implants with three different implant shoulder (neck) designs: a 1.5 mm machined implant neck (Replace Select Tapered, Nobel Biocare AB, Göteborg, Sweden), a rough implant neck with grooves (NobelReplace Tapered Groovy, Nobel Biocare AB), and a scalloped rough implant neck with grooves (NobelPerfect Groovy, Nobel Biocare AB). Although there was a statistically significant difference in MBL between different implant shoulder designs (smooth neck 1.19 ± 0.82 mm, rough neck 0.90 ± 0.57 mm, and scalloped neck 2.01 ± 0.77 mm), there were no differences between groups regarding the PES/WES outcomes, as well as patient satisfaction. In a prospective comparative study, Khraisat et al. [25] evaluated MBL and soft tissues around single implants with the scalloped shoulder design (Nobel Perfect, Nobel Biocare) and a smooth collar of 1.5 mm, within 3 years of function. The mean MBLs around scalloped implants were compared to MBLs around conventional flat platform 3.75 mm diameter TiUnite surface implants with external hex (MK III RP, Nobel Biocare), after both 1 and 3 years of function. The results of the present prospective study demonstrated that scalloped implants did not maintain marginal bone levels. All of the data are reported in Table 2.

Data from other reviews provide insufficient evidence about the efficacy of scalloped implant designs in the stability of peri-implant tissues [30, 31]. On the other hand, favorable results regarding scalloped implants were reported by Prasad et al. [29].

### 3.6. One-Piece versus Two-Piece Scalloped Implants

Consecutively, restored one-piece (NobelPerfect One-Piece) and two-piece (NobelPerfect, Nobel Biocare) scalloped dental implants were radiographically and clinically compared in a study of McAllister [26]. Radiographic evaluation of 16 two-piece scalloped implants and 9 one-piece scalloped implants revealed enhanced interproximal bone levels compared to a nonscalloped conventional flat-top implant design. Based on the Jemt system for interproximal soft tissue level evaluation, 78% of the two-piece implants scored 3 and 22% scored 2 and 89% of the one-piece implants scored 3 and 11% scored 2. The authors concluded that enhanced interproximal tissue preservation from scalloped implant designs may lead to more predictable aesthetic dental implant restorations in the anterior maxilla. All of the data are reported in Table 2.

### 3.7. Sloped Implants (MBL and BR)

No comparative studies reporting data on sloped implants were found that fulfilled the inclusion and exclusion criteria of this systematic review.

### 3.8. Aesthetic Outcomes (Papilla Index, PES, and WES) and Patient Satisfaction

Four studies reported no differences in aesthetic outcomes between scalloped and flat implants [19–22]. Tymstra et al. [22] and Van Nimwegen et al. [19] evaluated the soft tissues around the adjacent implants and the neighbouring teeth using the Papilla Index according to Jemt [32]. den Hartog et al. [20, 21] analyzed the volume of the interproximal papilla using the Papilla Index in the first study [20] and two objective aesthetic indexes, pink aesthetic score/white aesthetic score (PES/WES) and implant crown aesthetic index (ICAI), in the second study [21]. Three of them reported outcomes on patient satisfaction, using the patient questionnaire or VAS, scoring no differences between groups [19, 21, 22]. All the data are reported in Table 2.

## 4. Discussion

The aim of this systematic review was to identify whether there is a relationship between different implant shoulder positions/orientations/designs in the anterior dentition and prosthetic and/or implant failures, biological or mechanical complications, radiographic marginal bone loss, peri-implant buccal recession, aesthetic scores, and patient satisfaction after a minimum of 1-year function. The types of the implant analyzed were one-piece implants, compared with two-piece implants, and scalloped and sloped implants, compared with the conventional implant neck architecture (flat implants with the same level on 360°).

The results of the present systematic review indicate that different implant shoulder positions (scalloped, sloped, and one piece) seem to offer no benefit when compared to conventional, two-piece, flat implants.

A trend of higher implant failure and prosthetic complications were experienced in the one-piece group compared to the two-piece group, even if no statistically significant differences were found. This is in agreement with two systematic reviews by the same author [27, 28] which concluded that, despite high long-term prosthetic survival rates, technical and biological complications are frequent in one-piece implants independently by the loading protocols, implant surfaces, or type of edentulism.

One-piece implants are generally placed in a nonsubmerged approach. This means that implant placement is performed in a single surgical procedure, with no need for surgical reopening. Compared to a two-stage procedure, this approach is more comfortable for the patient, due to the fewer number of surgeries, and reduces the healing period. Implant shoulder placed at the level of the soft tissue offers many advantages since it is easily accessible for procedures such as impression taking and represents an excellent basis for cemented implant restorations [17, 18]. Moreover, due to its design, one-piece implants have their transmucosal surface unaltered during all of the prosthetic procedures since abutment reconnection is avoided (one-piece, one-part implants) or it is performed at the supramucosa or marginal mucosa level (one-piece, two-part implants). This avoids trauma to the soft tissue, which could result in a more apical position of the connective tissue and be responsible for additional marginal bone resorption.

A clinical study by Heijdenrijk et al. [9] evaluated the feasibility of using a two-piece implant system in a nonsubmerged procedure compared to the two-piece implant system placed in the traditional submerged procedure and one-piece implants placed in a nonsubmerged procedure. After 5 years of functioning, no association was found between the level of the microgap and the amount of bone loss, suggesting that two-piece implants used in a nonsubmerged procedure may be as predictable as when used in a submerged procedure or as one-piece implants [9].

Three studies included in this review reported differences in MBL between one- and two-piece implants. Thoma et al. [18] and Duda et al. [24] reported higher MBL in the one-piece implants, whilst Ormianer et al. [23] reported no differences between groups. Sanz Martin et al. [17] assessed the volumetric changes of the buccal soft tissue between baseline and 1 year of loading between one- and two-piece dental implants. This research failed to find significant differences between the one- and two-piece implant types with regard to tissue thickness, crown height, and facial tissue volume.

The concept of scalloped implants was introduced to maintain the alveolar ridge and the peri-implant soft tissue contour by mimicking the scalloped shape of natural topography of the healthy marginal bone contour [12]. The long-term results showed stable soft tissues around the scalloped implants in spite of some loss of marginal bone support in relation to the originally intended marginal bone level [33].

The primary aesthetic goal of the scalloped implant design is to avoid the dark, triangular space known as the ‘‘black triangle.” This space appears when bone remodeling results in loss of osseous support for the papillae [34]. The aesthetic concern is increased when a patient presents an alveolar morphotype, with a pronounced scalloped profile of the hard and soft tissues. This can be further complicated by the gingival display of a high smile line [34]. A five-year randomized controlled trial [19] was realized as follow-up of a 1-year report [22] to evaluate peri-implant soft and hard tissue of two adjacent implant-support restorations in the aesthetic region using a scalloped or flat platform. More bone loss and compromised interimplant papilla regeneration were obtained around scalloped implants, indicating that scalloped implants do not offer benefits in the aesthetic region [19, 22]. The other articles included in this review [20, 21, 25] comparing scalloped implants with the conventional implant neck architecture reported higher marginal bone loss in the test group (scalloped implants) compared to implants with the same level on 360°. Other than MBL, Khraisat [25] also analyzed the soft tissue level around scalloped single implants compared to conventional rough surface implants with external hex in the aesthetic zone over a 3-year period. The Jemt system was used to clinically assess sizes of the mesial and distal interproximal papillae, showing that soft tissue height was not consistently maintained around the scalloped area of the implants. Different results were obtained in a study of McAllister [26] where consecutively restored one- and two-piece scalloped implants were radiographically and clinically compared to a flat-top implant with similar implant geometry regarding taper and thread design. Enhanced interproximal hard and soft tissue preservation was obtained for scalloped implants leading to predictable aesthetic restorations in the anterior maxilla. The authors concluded that interproximal soft tissue levels may be enhanced by maintaining the crestal bone level and avoiding interproximal soft tissue attachment manipulation during abutment connection.

No comparative studies were found that fulfilled the inclusion and exclusion criteria of the sloped implants' systematic review. Nevertheless, the available data provide encouraging results for sloped implants in preserving the bone crest and the interplant papilla [13]. Placing an implant in a healed alveolar ridge with differences in height between the buccal and lingual bone crest will not allow the buccal part of the marginal portion of the implant to be completely invested in the bone, resulting in a risk of aesthetic complications [13]. In a prospective multicenter study, nonsubmerged implants (OsseoSpeed Profile implants; Astra Tech AB, Molndal, Sweden) were placed in a recipient site with a buccal-lingual bone height discrepancy of 2.0–5.0 mm, and the sloped part of the device was located at the buccal and most apical position of the preparation. Sixteen weeks later, bone level alterations were 0.02 mm lingually and 0.30 mm buccally, and at the 1-year reexamination, the average change in interproximal bone levels was 0.54 mm. The authors concluded that sloped implant placement in an alveolar ridge with a sloped marginal configuration resulted in minor remodeling, preserving discrepancies between buccal and lingual bone levels [13].

## 5. Conclusions

- Although no statistically significant differences were found, a trend of higher implant failure and prosthetic complications were experienced in the one-piece group compared to the two-piece group.
- A trend of higher marginal bone loss was found in the test group compared to the control group. This trend is moderate when comparing one-piece versus two-piece implants and high when comparing scalloped versus flat implants.
- Although no studies were found comparing sloped versus flat implants, preliminary results may encourage future studies.
- No differences were experienced between groups regarding aesthetic outcomes and patient satisfaction.

There was sufficient evidence that different implant shoulder positions/orientations/designs (scalloped, sloped, and one piece) offer no benefit when compared to two-piece, conventional flat implants. Current evidence is limited due to the quality of available studies. Marginal bone loss seems to be affected by the implant neck design, while aesthetics and patient satisfaction appear to be independent. Further studies, designed as randomized controlled clinical trials reported according to the CONSORT statement, are needed.

## Figures and Tables

**Table 1:** Reporting quality of all selected full-text articles.

Author and year	Implant shoulder design	Study characteristics				Selection bias		Performance bias	Detection bias	Attrition bias	Reporting bias		
		Study design	Follow-up	CONSORT/STROBE	Ethical board approval	Random sequence generation	Allocation concealment	Blinding of participants and personnel	Blinding of outcome assessors	Incomplete outcome data	Selective reporting	Other sources of bias	Overall risk of bias
Heijdenrijk et al. 2006 [9]	Scalloped	RCT	5 years	No	No	Unclear	Unclear	No	No	Medium risk	Low risk	Low risk	Medium risk
Sanz Martin et al. 2015 [17]^∗^	One piece	RCT	1 year	No	Yes	Computer-generated list	No	No	Unclear	Low risk	Low risk	Low risk	Medium risk
Thoma et al. 2014 [18]^∗^	One piece	RCT	1 year	No	Yes	Computer-generated list	No	No	No	Low risk	Low risk	Low risk	Medium risk
Van Nimwegen et al. 2015 [19]^∗^	Scalloped	RCT	5 years	Unclear	No	Unclear	No	No	No	Low risk	Low risk	Low risk	High risk
den Hartog et al. 2011 [20]^∗^	Scalloped	RCT	1 year	No	Yes	Randomization by minimization	Yes	No	No	Low risk	Low risk	Low risk	Medium risk
den Hartog et al. 2013 [21]^∗^	Scalloped	RCT	1 year	No	Yes	Randomization by minimization	Yes	No	No	Low risk	Low risk	Low risk	Medium risk
Tymstra et al. 2011 [22]^∗^	Scalloped	RCT	1 year	Unclear	No	Unclear	No	No	No	Low risk	Low risk	Low risk	High risk
Ormianer et al. 2016 [23]	One piece	Retrospective	5 years	No	No	Arbitrary	No	No	No	Low risk	Medium risk	Medium risk	High risk
Duda et al. 2016 [24]	One piece	Retrospective	3 years	No	No	No	No	No	No	Low risk	Medium risk	Medium risk	High risk
Khraisat et al. 2013 [25]	Scalloped	Prospective	3 years	No	Yes	No	No	No	No	Low risk	Low risk	Low risk	Medium risk
McAllister 2007 [26]	Scalloped/one piece	Prospective	4 to 28 months	No	No	No	No	No	No	Medium risk	Medium risk	Medium risk	High risk

^∗^Manuscripts that included the same cohort of patients.

**Table 2:** Results of the included studies.

Author and year		Patients/implants	Follow-up	Failed implants	Complications	MBL (mm)	BR	Papilla Index	PES/WES	Patient questionnaire/VAS
*One-piece (test, T) versus two-piece (control, C) implants*								
Heijdenrijk et al. 2006 [9]	T	20/40	5 years	0	NR	1.8	NR	NR	NR	NR
	C	20/40	5 years	1	NR	1.6	NR	NR	NR	NR
	C	20/40	5 years	1	NR	1.4	NR	NR	NR	NR
Sanz Martin et al. 2015 [17]^∗^	T	30/65	1 year	NR	NR	NR	CHC: −0.17; VC: −0.03	NR	NR	NR
	C	30/86	1 year	NR	NR	NR	CHC: 0.02; VC: −0.12	NR	NR	NR
Thoma et al. 2014 [18]^∗^	T	30/65	1 year	1	NR	0.27	NR	NR	NR	NR
	C	30/86	1 year	0	NR	0.05	NR	NR	NR	NR
Ormianer et al. 2016 [23]	T	24/34	5 years	0	4^ç^	NR	NR	NR	NR	NR
	C	24/38	5 years	0	8^ç^	NR	NR	NR	NR	NR
Duda et al. 2016 [24]	T	13/49^§^	5 years	7	NR	1.45	NR	NR	NR	NR
	T	11/24°	5 years	2	NR	1.71	NR	NR	NR	NR
	C	10/39	3 years	0	NR	0.9	NR	NR	NR	NR

*One-piece (test, T) versus two-piece (control, C) scalloped implants*								
McAllister 2007 [26]	T	9/9	18 months	0	NR	NR	NR	16 : 3; 2 : 2	NR	NR
	T	13/16	28 months	0	NR	NR	NR	25 : 3; 7 : 2	NR	NR
	C	NR/12	12 months	0	NR	NR	NR	NR	NR	NR

*Scalloped (test, T) versus flat (control, C) implants*								
Van Nimwegen et al. 2015 [19]^∗^	T	20/40	5 years	2	2	3.4/2.4	NR	16, 32	NR	8.4
	C	20/40	5 years	0	0	1.5/1.3	NR	19, 38	NR	9.1
den Hartog et al. 2011 [20]^∗^	T	31/31	18 months	0	NR	2.01	0.25	36 : 3; 41 : 2; 23 : 1	NR	NR
	C	31/31	18 months	1	NR	1.19	0.18	31 : 3; 53 : 2; 16 : 1	NR	NR
	C	31/31	18 months	0	NR	0.9	0.28	34 : 3; 45 : 2; 19 : 1	NR	NR
den Hartog et al. 2013 [21]^∗^	T	31/31	18 months	0	NR	2.01	NR	NR	6.6/7.2	9.1
	C	31/31	18 months	1	NR	1.19	NR	NR	6.0/7.2	8.8
	C	31/31	18 months	0	NR	0.9	NR	NR	6.3/7.4	8.9
Tymstra et al. 2011 [22]^∗^	T	20/40	1 year	0	NR	2.7/2.6	0.3	19, 38	NR	8.6
	C	20/40	1 year	0	NR	0.9	0.1	19, 38	NR	8.3
Khraisat et al. 2013 [25]	T	12/12	3 years	NR	NR	3.48/3.52	NR	NR	NR	NR
	C	12/12	3 years	NR	NR	1.35/1.27	NR	NR	NR	NR

^∗^Manuscripts that included the same cohort of patients; ^ç^porcelain fractures; ^§^immediately loaded one-piece; °delayed loaded one-piece; NR: not reported; CHC: crown height changes in mm; VC: volume changes in mm.
